# The effect of tailored Web-based interventions on pain in adults: a systematic review protocol

**DOI:** 10.1186/s13643-016-0233-5

**Published:** 2016-04-12

**Authors:** Géraldine Martorella, C. Gélinas, M. Bérubé, M. Boitor, S. Fredericks, S. LeMay

**Affiliations:** TMH Center for Research and Evidence-Based Practice, College of Nursing, Florida State University, 104F Vivian M. Duxbury Hall, 98 Varsity Way, Tallahassee, FL 32306 USA; Quebec Nursing Intervention Research Network (RRISIQ), Montreal, Canada; Ingram School of Nursing, McGill University, Montreal, Canada; Centre for Nursing Research and Lady Davis Institute, Jewish General Hospital, Montreal, Canada; Alan Edwards Centre for Research on Pain, McGill University, Montreal, Canada; Daphne Cockwell School of Nursing, Ryerson University, Toronto, Canada; Faculté des Sciences infirmières, Université de Montréal, Montreal, Canada; Centre de Recherche du CHU Ste Justine, Montreal, Canada

**Keywords:** Web-based, Internet-based, Tailored, Pain, Adults, Systematic review

## Abstract

**Background:**

Information technologies can facilitate the implementation of health interventions, especially in the case of widespread conditions such as pain. Tailored Web-based interventions have been recognized for health behavior change among diverse populations. However, none of the systematic reviews looking at Web-based interventions for pain management has specifically addressed the contribution of tailoring.

**Methods:**

The aims of this systematic review are to assess the effect of tailored Web-based pain management interventions on pain intensity and physical and psychological functions. Randomized controlled trials including adults suffering from any type of pain and involving Web-based interventions for pain management, using at least one of the three tailoring strategies (personalization, feedback, or adaptation), will be considered. The following types of comparisons will be carried out: tailored Web-based intervention with (1) usual care (passive control group), (2) face-to-face intervention, and (3) standardized Web-based intervention. The primary outcome will be pain intensity measured using a self-report measure such as the numeric rating scale (e.g., 0–10) or visual analog scale (e.g., 0–100). Secondary outcomes will include pain interference with activities and psychological well-being. A systematic review of English and French articles using MEDLINE, Embase, CINAHL, PsycINFO, Web of Science, and Cochrane Library will be conducted from January 2000 to December 2015. Eligibility assessment will be performed independently in an unblinded standardized manner by two reviewers. Extracted data will include the following: sample size, demographics, dropout rate, number and type of study groups, type of pain, inclusion and exclusion criteria, study setting, type of Web-based intervention, tailoring strategy, comparator, type of pain intensity measure, pain-related disability and psychological well-being outcomes, and times of measurement. Disagreements between reviewers at the full-text level will be resolved by consulting a third reviewer, a senior researcher.

**Discussion:**

This systematic review is the first one looking at the specific ingredients and effects of tailored and Web-based interventions for pain management. Results of this systematic review could contribute to a better understanding of the mechanisms by which Web-based interventions could be helpful for people facing pain problems.

**Systematic review registration:**

PROSPERO CRD42015027669

**Electronic supplementary material:**

The online version of this article (doi:10.1186/s13643-016-0233-5) contains supplementary material, which is available to authorized users.

## Background

Unrelieved pain and suffering are a considerable public health issue although efforts have been made to recognize pain management as a human right [1, 2]. Cognitive behavioral therapy (CBT) interventions have been widely used to manage chronic pain, and their impact on pain is well established through a multitude of randomized controlled trials across diverse populations [3, 4]. In regard to acute pain, particularly postoperative pain, educational interventions have been the most frequently studied and helpful approaches for about 30 years [5–9]. Nonetheless, both for acute and chronic pain management, significant barriers such as time, cost, and distance generate considerable treatment accessibility issues and inhibit the improvement of pain management, allowing for other formats of interventions other than face to face to be implemented [3, 10–12]. Computers and information technologies have been part of our lifestyle for some time, and they can facilitate the implementation of health interventions, especially in the case of widespread conditions such as pain.

The influence of Web-based interventions, as opposed to non-Web-based, on health behavior change has started to be demonstrated in the last decade [13]. However, only a few systematic reviews looking at the ability of Internet or Web-based interventions to influence health behavior change in regard to pain management have been conducted [14–16]. The authors concluded that the results were promising in terms of pain reduction and functional and emotional well-being improvement. They also underlined that it is still unknown what patient clienteles benefit most from this approach [14, 15]. Among Internet or Web-based interventions, various approaches have been tested. Two meta-analyses studied the particular contribution of Web-based CBT and psychotherapies [15, 17]. A small positive effect was found on pain, and results remain unclear considering the high dropout rates and the heterogeneity related to assessments used (measures and timing), type of pain-related diseases, and interventions (i.e., content, format, dose) but also the lack of diversity in patients (e.g., mainly women, Caucasian, college educated) [3, 14, 15]. The authors also suggested that further research is needed in order to know which patients would be the best responders to Web-based CBT in terms of pain relief. Nonetheless, none of the systematic reviews looking at Web-based interventions for pain management has specifically addressed the contribution of tailoring ingredients.

Experts in health behavior change have shown that conveying health information without considering individual differences may inhibit behavior change [18–24]. Tailoring strategies respond to this concern. Moreover, face-to-face CBT for pain management has been tailored to fit a variety of clienteles [3]. Tailoring is defined as a process for creating individualized communications using personal data related to health outcomes in order to meet individual needs [19, 21, 23, 25, 26]. Three mechanisms have been highlighted [19, 27]. Firstly, personalization refers to the inclusion of specific and personally identifiable information within the content (e.g., names, age, or specific behaviors) gathered during the assessment phase. Personalization helps increase the perceived meaningfulness of the message by creating the impression that the message was designed specifically for the individual [19]. Secondly, feedback refers to individual recommendations based on an expert assessment of the individual’s needs or characteristics related to the targeted behaviors [19]. Feedback directs the attention of the individual to their own characteristics or behaviors (which are determined during assessment) that they need to address, improve, or change. The key to providing feedback is referring to how the individual answered certain assessment questions within the tailored message (e.g., “It seems from your responses that you believe that…”), evaluating this response, and then providing individualized feedback. The final technique, adaptation, or content matching, refers to creating content packages that are pertinent to an individual and selected based on known determinants of the targeted behavior [19]. Adaptation requires analyzing individual responses, determining what types of messages would be most effective for specific individuals, and then matching the appropriate content to each individual. Examples of variables used to adapt messages include the following: pain intensity, self-efficacy, motivation, beliefs, employment status, and cultural values. Table 1 provides a summary of tailoring ingredients previously found in tailored Web-based interventions [27].

**Table 1:** Ingredients of tailored Web-based interventions [27]

Tailoring criteria	Tailoring mechanism	Level of tailoring
• Information needs (e.g., pain beliefs) • Risk factors (e.g., pain level) • Health behaviors (e.g., self-efficacy) • Stages of change or other theoretical underpinnings	• Personalization • Feedback • Adaptation/content matching	• Online assessment and tailored feedback • Online assessment, tailored feedback, and content • Customized health program including goal setting and monitoring

Clinically relevant results and statistically significant effect sizes of tailored Web-based interventions have been recognized for health behavior change among diverse populations [18, 27, 28]. However, the tailored content of Web-based interventions for pain management has not been described and its specific effect on pain has not been evaluated in a systematic review.

### Objectives

Considering the contribution of tailored Web-based interventions in other health fields and the lack of knowledge on these approaches regarding pain management, we plan to conduct a systematic review to answer the following research questions:

- What is the effect of tailored Web-based pain management interventions for adults compared to usual care, face-to-face interventions, and standardized Web-based interventions on
  - ○ Pain intensity?
  - ○ Physical function?
  - ○ Psychological function?

## Methods/design

This systematic review has been developed based on Preferred Reporting Items for Systematic Reviews and Meta-Analyses (PRISMA) guidelines for reporting systematic reviews evaluating health care interventions [29–31]. A PICOS question (participants, interventions, comparisons, outcomes, study designs) was developed to identify criteria for study inclusion as follows:

- Types of participants
  Patients of 18 years of age or more and suffering from any type of pain (acute, chronic) will be selected. Pediatric patients, being a specific population, will not be included as it may increase heterogeneity in the design and content of interventions, and the latter usually involves parents in the therapy. Moreover, it seems difficult to conduct subgroup analysis because of the small sample sizes found in pediatric studies [15].

- Types of interventions
  Web-based interventions for pain management, available online or not and including at least one of the three tailoring strategies (personalization, feedback, or adaptation) [19, 27], will be selected. Tailored interventions could typically include educational, behavioral, cognitive, CBT, and psychological support approaches [14]. Web modalities can include the following: educational Web sites, online support groups, online cognitive behavioral therapy programs, email, discussion board, chat, short messaging system, E-journal, newsletters, animation, audio narration, online quizzes, and games [14, 27]. Regarding the dose (frequency and duration) of the Web-based intervention, chronic pain management interventions can last from 4 up to 20 weeks and involve from four to eight sessions [14, 15]. As for acute pain management interventions, they do not typically include a number of sessions but an access to a Web site before and after a surgery or before and after a procedure [14]. The type of contact with a therapist includes email, telephone, and Internet meeting [15].

- Types of comparisons
  According to previous findings, eligible comparators will include the following: (1) standard or usual care (i.e., passive control group receiving usual medical and nursing care in hospital settings, including booklet, or outpatients receiving medical follow-up in clinics or on a pain clinic waitlist); (2) face-to-face educational or psychological (e.g., CBT, cognitive, behavioral, support) interventions; and (3) Web-based standardized intervention (e.g., Web site consultation, Web-based standardized education, standardized emails) [14, 15].

- Types of outcomes
  Outcomes were selected according to the Initiative on Methods, Measurement, and Pain Assessment in Clinical Trials (IMMPACT) recommendations [32, 33]. Six core domains were identified by the IMMPACT: (1) pain, (2) physical functioning, (3) emotional functioning, (4) participant ratings of improvement and satisfaction with treatment, (5) symptoms, and (6) participant disposition. Primary and secondary outcomes were selected according to their association to pain intensity and the relative homogeneity found in their measures.
  Primary outcome addresses the first domain of pain outcomes and will include pain intensity measured using a self-report measure such as the numeric rating scale (e.g., 0–10) or visual analog scale (e.g., 0–100). Pain intensity is therefore a mandatory outcome for the study to be included in this systematic review.
  Secondary outcomes address the second and third domains and will include pain-related disability (e.g., Brief Pain Inventory (BPI), return-to-work Multidimensional Pain Inventory (MPI), Pain Disability Index (PDI), Roland Morris Disability Questionnaire (RMDQ)) and psychological well-being (e.g., Hospital Anxiety Depression Scale (HADS), Pain Catastrophizing Scale (PCS), Brief Pain Inventory (BPI), Beck Depression Inventory (BDI), health-related quality of life (HRQoL)).
  The timeline of outcomes for acute pain management interventions will include measures after completion of the intervention, between the first day after surgery and hospital discharge, and/or medical follow-up appointment. The timeline of outcomes for chronic pain management interventions will include measures before and after treatment.

- Type of study designs
  Randomized controlled trials (RCTs) will be included in this systematic review.

### Data collection and analysis

A systematic review of English and French articles using MEDLINE, Embase, CINAHL, PsycINFO, Web of Science, and Cochrane Library will be conducted from January 2000 to December 2015. 2000 was chosen as a cut-off date considering the large number of RCTs on Web-based interventions published in the last decade. Reviewing the reference lists of relevant articles and narrative reviews will identify supplementary articles. The primary author has developed the search strategy with an experienced research librarian. An electronic search using subject headings for each database and keywords to avoid missing non-indexed concepts will be conducted.

Search terms will include the following: pain, pain management, program, intervention, Internet, Internet-based, Online, Web-based, and Mobile or Mobile applications. Table 2 illustrates the search strategy used in Embase and suitable for other databases.

**Table 2:** Search strategy used in Embase

1996 to 2015 week 52	
No.	Search statement
1	Internet/or web-based.mp.
2	Internet-based.mp.
3	Online.mp.
4	Mobile Applications/ or mobile.mp.
5	Intervention.mp.
6	Interventions.mp.
7	Program*.mp.
8	Pain/
9	Pain Management/
10	1 or 2 or 3 or 4
11	5 or 6 or 7
12	8 or 9
13	10 and 11
14	12 and 13
15	Limit 14 to (yr="2000 -Current" and (adult <18 to 64 years> or aged <65+ years>))

### Selection of studies

Manuscripts in English or French published in a peer-reviewed journal and reporting results of randomized controlled trials involving adult patients (18 years and older), of any sex, and with pain of any kind (acute, i.e., <3 months; chronic, i.e., 3–6 months and beyond [34]) will be selected. Eligibility assessment will be performed independently in an unblinded standardized manner by two team members (GM, CG). Firstly, titles and abstracts will be screened. If a trial is potentially eligible, the full text will be reviewed. The two reviewers are researchers in the field of pain with clinical background. Disagreements between the reviewers at the full-text level will be resolved by consulting a third reviewer, a senior researcher (SL).

### Data extraction and management

Data will be extracted independently by two reviewers (two doctoral students in pain intervention research with clinical background; MBe, MBo) using a predefined data extraction form. The data extraction form will be developed based on the Cochrane Consumers and Communication Review Group’s data extraction template [35] and will be pilot tested and refined accordingly. Disagreements will be resolved by discussion between the two reviewers, and if consensus is not reached, a third reviewer will be involved (GM or CG). Inter-rater reliability (intra-class correlation coefficient or kappa coefficient) will be assessed to demonstrate consistency in data selection and extraction. Missing data, particularly related to interventions, will be requested from authors in order to be able to determine the tailoring components. Extracted data will include the following: sample size, sample demographics, dropout rate, number and type of study groups, type of pain (acute vs. chronic), inclusion and exclusion criteria, study setting, type of Web-based intervention (i.e., setting, mode, dose, contact with therapist), tailoring strategy (i.e., personalization, feedback, adaptation), comparator (i.e., passive control group vs. active control group), type of pain intensity measure, pain-related disability and psychological well-being outcomes, and times of measurement. The software DistillerSR™ will be used to facilitate data extraction and management. The data will be presented in summary tables and figures.

### Quality assessment

The quality of the included randomized controlled trials will be assessed through the evaluation of the risk of bias at both the study level and outcome level (i.e., pain intensity). The two reviewers involved in data extraction (MBe, MBo) will use the well-established Cochrane Collaboration’s tool for assessing the risk of bias in randomized trials [36, 37]. Although its usability may need improvements, it is still the most recommended tool [38]. It covers six domains: selection bias, performance bias, detection bias, attrition bias, reporting bias, and other bias. Judgment of whether high, low, or unclear risk of bias will be made [37]. Any variation in the assessment will be discussed.

### Data synthesis

The PRISMA framework will be used to ensure the transparent reporting of this systematic review including the flow diagram [39] (see Additional file 1 for the PRISMA-P checklist). A narrative review of the findings from the eligible studies will be provided including the tailoring content of interventions. Descriptive statistics will be used to (1) delineate the characteristics of the studies included, (2) describe the characteristics of individuals comprising the samples, and (3) describe the approach, mode, and dose of tailored Web-based interventions associated with statistically significant changes in pain intensity.

In regard to the primary outcome, continuous pain intensity outcomes will be converted to the 0–100 scale. An independent-sample *t* test will be conducted to determine whether or not a statistically significant difference in pain intensity (i.e., pre/post intervention mean change for chronic pain patients and post intervention mean score for acute pain patients) exists between groups (i.e., tailored Web-based intervention vs. eligible comparator). The mean difference (MD), or standardized mean difference (SMD) in the case of different scales, with 95 % confidence intervals (CI) will help to measure the treatment effect. As for secondary outcomes, MD and/or risk ratios (RR) with their respective 95 % CI will be used depending on the type of variables, i.e., continuous vs. dichotomous.

### Assessment of heterogeneity

Heterogeneity of studies will be evaluated using the chi-square test for heterogeneity and the *I*-squared test. If less than 50 %, studies will be considered sufficiently homogeneous to proceed with a meta-analysis [40]. The meta-analysis will include studies with low risk of bias [37]. However, considering previous findings in terms of variety of pain, patient populations, and outcome measures [14], the possibility of conducting a meta-analysis seems unlikely.

### Assessment of reporting biases

Publication bias will also be evaluated using funnel plot analyses of asymmetry [41]. The possible reasons for asymmetry will be investigated [42, 43].

### Subgroup analysis

Subgroup analyses will be performed according to the type of comparator (passive control group, i.e., usual/standard care, vs. active control group, i.e., face-to-face intervention or standardized Web-based intervention) and the type of pain as well (acute pain vs. chronic pain).

### Sensitivity analysis

The quality and strength of the evidence regarding the most important outcome (i.e., pain intensity) will be summarized by using the Grades of Recommendations, Assessment, Development and Evaluation (GRADE) approach [37, 44].

## Discussion

Web-based interventions, particularly tailored ones, have been shown to impact health behaviors and improve health outcomes among various populations [18, 28]. Web-based interventions have been found to be promising for individuals suffering from pain [14]. However, small effects on pain have been shown [14, 15]. Considering the variety of pain conditions and people facing them, tailored approaches seem to be an interesting avenue.

This systematic review is the first one looking at the specific ingredients and effects of tailored and Web-based interventions for pain management. Results of this systematic review could contribute to a better understanding of the mechanisms by which Web-based interventions could be helpful for people facing pain problems. It is also a starting point in exploring what types of patients could benefit most from these approaches. The findings of this study can enlighten specific elements to allow the development of pain management interventions.

## Additional file

Additional file 1: PRISMA-P 2015 checklist. The PRISMA framework will be used to ensure the transparent reporting of this systematic review including the flow diagram. (DOCX 35.5 kb)

## Abbreviations

CBT: cognitive behavioral therapy
CINAHL: Cumulative Index to Nursing and Allied Health Literature
IMMPACT: Initiative on Methods, Measurement, and Pain Assessment in Clinical Trials
PICOS: participants, interventions, comparisons, outcomes and study designs
PRISMA: Preferred Reporting Items for Systematic Reviews and Meta-Analyses
